# Acute changes in handgrip strength, lung function and health-related quality of life following cardiac surgery

**DOI:** 10.1371/journal.pone.0263683

**Published:** 2022-02-23

**Authors:** Nnamdi Mgbemena, Anne Jones, Pankaj Saxena, Nicholas Ang, Siva Senthuran, Anthony Leicht

**Affiliations:** 1 Department of Physiotherapy, James Cook University, Townsville, Queensland, Australia; 2 Australian Institute of Tropical Health & Medicine, James Cook University, Townsville, Queensland, Australia; 3 Department of Cardiothoracic Surgery, Townsville University Hospital, Townsville, Queensland, Australia; 4 Department of Surgery, James Cook University, Townsville, Queensland, Australia; 5 Department of Intensive Care Medicine, Townsville University Hospital, Townsville, Queensland, Australia; 6 Department of Sports and Exercise Science, James Cook University, Townsville, Queensland, Australia; Universitaria di Bologna, ITALY

## Abstract

**Background:**

Handgrip strength (HGS), lung function and health-related quality of life (HRQoL) are relevant indicators of future cardiovascular risk and mortality. The impact of cardiac surgery on these predictive variables has been under-explored. The aim of this study was to determine the acute (within hospital) changes in HGS, lung function and HRQoL, and their relationships, in adults undergoing elective cardiac surgery. Further, the study examined the relationship between these variables and the predictors for lung function and HRQoL in these patients.

**Methods:**

The study was a prospective cohort study that involved 101 patients who completed pre-operative (1–2 days before surgery) and physiotherapy discharge (5–7 days after surgery) assessments. Handgrip strength, lung function and HRQoL were assessed using JAMAR dynamometers, Vitalograph-Alpha or EasyOne spirometer, and Short-Form 36 questionnaire, respectively. Changes in these variables and their relationships were analysed using paired t-test and Pearson correlation coefficients, respectively. Prediction of lung function and HRQoL using HGS and other co-variates was conducted using regression analysis.

**Results:**

At the time of physiotherapy discharge, lung function, HGS and the physical component of HRQoL were significantly (<0.001) reduced compared to their pre-operative values. Significant (<0.001) and moderate correlations were identified between HGS and lung function at pre-operation and physiotherapy discharge. Handgrip strength was a significant predictor of lung function pre-operatively but not at physiotherapy discharge. Pre-operative lung function and HRQoL, as well as other variables, were significant predictors of lung function and HRQoL during physiotherapy discharge.

**Conclusions:**

Undergoing cardiac surgery acutely and significantly reduced lung function, HGS and physical component of HRQoL in adults with cardiac disease. Assessment of HGS at physiotherapy discharge may be a poor indicator of operative changes in lung function and HRQoL. Clinicians may consider HGS as an inadequate tool in predicting lung function and HRQoL following cardiac surgery.

## Introduction

Cardiac diseases are the single, largest cause of death in the general population and have become a global public health concern [1] with cardiac surgery a primary treatment for cardiac disease management [2]. Following cardiac surgery there are variable changes in important indicators of musculoskeletal and cardiorespiratory function such as handgrip strength (HGS) and lung function/efficiency [3–6], most likely due to inflammation [7, 8]. This inflammatory deconditioning may lead to a concomitant decrease in health-related quality of life (HRQoL) for patients [3] with reduced HRQoL predictive of future cardiac disease risk and/or related deaths [9].

To date, studies of post-surgical changes in HGS, lung function or HRQoL for cardiac populations have focused on these indicators following hospital discharge (greater than one month) [3–5, 10]. The in-hospital recovery period has been reported to be a vulnerable time for patients following cardiac surgery [11] with a few studies examining patients at this time and reporting contradictory changes in HGS [6, 12]. For example, da Silva et al. [6] reported a significant reduction in HGS between pre-operative assessment and a day before discharge (14.8kgf vs. 11.5kgf; p<0.001), while Fu et al. [12] reported similar HGS between pre-operative and post-surgical (5-7days) values. Previous studies that investigated the acute impact of cardiac surgery on lung function reported marked reductions at two [13] and four [5] days following surgery with no study reported at the point of physiotherapy discharge from the hospital. To the best of our knowledge, no study has assessed the effect of cardiac surgery on HRQoL in these patients at the point of physiotherapy discharge (5–7 days after surgery). Identifying the amount of recovery for HGS, lung function and HRQoL at the point of physiotherapy discharge would help clinicians in understanding patient recovery prognosis and potentially, post-physiotherapy discharge destination and rehabilitation requirements [14].

Recently, a systematic review reported significant associations between HGS and lung function in healthy adults, while variable associations were reported in adults living with chronic diseases, and none reported in cardiac populations [15]. Confirmation of the association between HGS, lung function and HRQoL in cardiac surgical patients may support the predictive ability of an inexpensive tool like HGS for lung function and HRQoL in clinical practice. Therefore, the aim of this study was to examine the acute (within hospital) changes in these variables in patients undergoing cardiac surgery. Secondary aims were to examine the relationship between these variables, and to determine the ability of HGS to predict lung function and HRQoL, given the limited work to date for cardiac populations [15]. It was expected that assessment of HGS would provide clinicians with a simple tool to identify cardiac disease patient status and prognosis in terms of lung function and HRQoL.

## Methods

### Study design and setting

This study was a prospective, observational, cohort investigation conducted between January 2020 and April 2021 at two regional hospitals, Townsville University Hospital and the Mater Hospital Townsville. Assessments of HGS, lung function and HRQoL were conducted prior to cardiac surgery (baseline, 1–2 days before surgery) and at the time of physiotherapy discharge (5–7 days post-surgery). Patients were discharged from in-hospital physiotherapy management when deemed safe in terms of mobility and daily activities, were able to conduct breathing and airway clearance techniques, and manage independently at home [16]. All assessments were conducted by trained health professionals (e.g. physiotherapists, allied health assistants) using standardised procedures given the reported variability in assessment protocols of prior work [15]. All patients provided written informed consent prior to participation. Ethics and research governance approvals were obtained from the Human Research Ethics Committee, Townsville University Hospital (HREC/2019/QTHS/53274). The study was registered with the Australian New Zealand Clinical Trial Registry (ACTRN12619001515189).

### Participant recruitment and selection criteria

Patients meeting the following inclusion criteria were invited to participate in this study: aged 18 years or greater; ability to comprehend, read and speak English; diagnosed with cardiac disease and scheduled to undergo coronary artery by-pass graft (CABG), valvular replacements/repairs or combined surgeries at the hospitals. Patients meeting the following criteria were excluded from this study: undergoing emergency cardiac surgery; were pregnant; exhibited neuromuscular weakness as a result of a pre-existing condition; were involved in other clinical trials involving medications or interventions known to impact the variables of interest in the current study; and patients with diagnosed carpal tunnel syndrome, other upper limb injuries/deformities or had undergone hand surgery in the past three months.

### Research procedures and data collection

The following demographic and clinical characteristics for each patient were obtained from medical records to describe patients’ current health status and functional levels: age; sex; body mass index (BMI); ethnicity; smoking and alcohol drinking status; socio-economic status; physical activity level via self-reported question; New York Heart Association (NYHA) classification; left ventricular ejection fraction (LVEF); type of cardiac surgery to be undertaken; and risk of death assessment via Acute Physiology and Chronic Health Evaluation III score and the intensive care unit (ICU) derived, Australian and New Zealand Risk of Death.

Cardiac operative and post-operative characteristics, which have been shown to influence patients’ recovery [17], were obtained from participant’s medical records and included: aortic cross clamp time in minutes (ACXT); cardiopulmonary by-pass time in minutes (CBPT); ICU length of stay in days (ICULOS); discharge destination (i.e. home, aged care facility etc.); and total hospital length of stay in days. During physiotherapy discharge assessments, patient-reported chest pain was assessed using the numerical rating scale (0 = no pain to 10 = worst pain ever) at rest and during coughing, as poorer lung function has been associated with increased chest pain after cardiac surgery [13].

### Methods of assessment

#### Handgrip strength

Pre-operative assessment of HGS was conducted using a Jamar hydraulic hand dynamometer (Model 5030J1, Patterson Medical, Warrenville, IL) or Jamar hydraulic hand dynamometer (Model 5030J1, Performance Health, China) while all physiotherapy discharge assessments were conducted using the Jamar hydraulic hand dynamometer (Model 5030J1, Performance Health, China). Both hand dynamometers were available within a busy allied health department with each device calibrated annually as per the manufacturer’s recommendations. These assessments followed the guidelines of the American Society of Hand Therapists and included adjustment of the dynamometer handle to the second position with the patient seated without an arm-rest, and both hip and knee joints at 90^o^ while the feet rested flat on the ground [18]. While seated, patients’ shoulder joints were adducted, elbows flexed to 90^o^ with the forearms in a neutral position and the wrists in 15-30^o^ dorsiflexion and 0-15^o^ ulnar deviation [18]. Standardised instructions were provided to patients for all HGS assessments [19]. Patients completed three HGS trials for the dominant hand to achieve a grip phase of six seconds and a rest phase of at least 15 seconds per trial [19]. The highest value of the three trials was recorded as maximal HGS.

#### Lung function assessment

Pre-operative assessment of lung function was assessed using an EasyOne spirometer (Model 2001, ndd Medizintechnik, Switzerland) or Vitalograph Alpha 6000 (Vitalograph Ltd, Ireland) while all physiotherapy discharge assessments were conducted using the Vitalograph Alpha 6000 (Vitalograph Ltd, Ireland). Both spirometers were available within a busy allied health department with each device calibrated as recommended by the manufacturer. During the lung function assessments, patients were seated with the hip and knee joints at approximately 90°. Patients applied a nose clip, inhaled fully and rapidly, and then forcefully and maximally exhaled through a disposable mouthpiece with verbal encouragement [20] during each assessment. Three dynamic lung function indices, forced expiratory volume in one second (FEV_1_), forced vital capacity (FVC) and peak expiratory flow rate (PEFR) were obtained from a minimum of three repeatable and acceptable trials. Repeatability criteria were that the two highest values for FEV_1_ and FVC were within 0.15 L of each other while standardised acceptability criteria for FEV_1_ and FVC were also followed [20]. The highest value obtained for FEV_1_, FVC and PEFR were utilised in analyses [20]. Actual values, pre- operative to physiotherapy discharge changes ([physiotherapy discharge–pre-operative]/pre-operative x 100) of FEV_1_, FVC and PEFR, and percentage of predicted values were retrieved for analyses. Percentage of predicted values were calculated online (http://gli-calculator.ersnet.org/index.html) using equations incorporating patient’s height, age, sex and actual values of FEV_1_ and FVC.

Given that various devices available within a busy allied health department were utilised for assessments, inter-instrument reliability was examined separately with 30 healthy adults. The Vitalograph Alpha spirometer and the Jamar dynamometer model used during physiotherapy discharge assessments were deemed as the reference devices. Statistical differences between devices were noted (unpublished observations) and subsequently regression equations were developed to adjust all results of the non-reference devices [21] to ensure equivalent comparisons in the current study.

#### Health-related quality of life assessment

Self-reported HRQoL was assessed using the Short Form-36 medical outcome (SF-36) questionnaire [22], which has been validated in cardiac populations [23]. The SF-36 consists of 36 items that assess eight domains of HRQoL, with four domains contributing to a physical component summary (PCS) score and the remaining four domains contributing to a mental component summary (MCS) score [22]. Each domain and component summary score were normalised via Australian-based scoring algorithms [24]. Each score ranged between 0 and 100 with a higher value representing better HRQoL [22].

### Statistical analyses

Based on the study by da Silva et al. [6] that showed a change of 3.3kg in HGS and an effect size of 0.55, a minimum number of 71 patients (90% power, p<0.05) was needed to detect a significant change in HGS. Given that the patient drop-out rate was substantial (~38%) in a prior study [6], greater than 100 patients were recruited to meet the aims of this study. Normality of the data was checked via the Kolmogorov-Smirnov test and Lilliefors correction with the properties of the central limit theorem [25] applied to engage parametric statistical analyses. Changes in HGS, lung function (FEV_1_, FVC and PEFR) and HRQoL (PCS and MCS) due to surgery (i.e. pre-operative vs. physiotherapy discharge) were examined via paired t-tests. Relationships between variables were identified via Pearson correlation coefficients. Correlation coefficients categorised relationships as weak (0.00–0.30), moderate (0.31–0.70) and strong (>0.7) [26]. Predictive ability of HGS and demographic/operative characteristics (e.g. age, sex, weight, height, ethnicity, NYHA class, LVEF ACXT, CBPT, ICULOS, chest pain during coughing and comorbidities) for lung function and HRQoL, including operative-induced changes, was determined via stepwise multiple regression analysis. All results were presented as frequency or mean (standard deviation) with the level of significance set at <0.05. All analyses were conducted using the Statistical Package for the Social Sciences, version 27.0 (IBM Inc, Chicago IL).

## Results

### Demographic characteristics

One hundred and twenty adults were recruited for this study while 101 patients (81% males) completed both pre-operative and physiotherapy discharge assessments for HGS, lung function and HRQoL. Reasons for patient withdrawal or loss of data were death (n = 3), inter-regional hospital transfer (n = 7) and physiotherapy discharge assessments not recorded (n = 9). Patients’ demographic and operative characteristics are presented in Table 1 with most of these similar (p>0.05) to patients who withdrew or did not have pre-operative and physiotherapy discharge assessments recorded. The majority of patients were senior (≥65 years), retired, non-indigenous, overweight males with a NYHA classification of I and II. Overall, the most prevalent comorbidities were hypertension, dyslipidaemia, type II diabetes mellitus and obesity, with 80% of the patients undergoing isolated CABG surgery (Table 1).

**Table 1:** Demographic and peri-operative characteristics of participants (n = 101 unless otherwise stated).

Variables	All
Sex (males)^a^	82(81%)
Age (years)^b^	64.6(11.3)
Height (m)^b^	1.71(0.09)
Weight (kg)^b^	88.3(21.5)
Body Mass Index (kg/m^2^)^b^	29.99 (6.50)
Ethnicity^a^	
Indigenous/Non-indigenous/Others	15/78/8
Smoking status^a^	
Non-smoker/Ex-smoker/Smoker	36/47/17
Alcohol consumption status^a^	
Non-drinker/Ex-drinker and Drinker	14/17/69
Highest educational level^a^	
Primary/High-school/Tertiary	3/81/17
Physical activity level (n = 96)^a^	
Active/Inactive	93/3
Employment status^a^	
Employed/Unemployed/Retired	33/9/59
Hand Dominance^a^	
Right/Left/Ambidextrous	89/11/1
Type of surgery^a^	
Isolated CABG/Isolated AVR/Isolated MVR/CABG+AVR or MVR	80/12/4/5
NYHA (n = 100)^a^	
I/II/III/IV	47/30/23/0
APACHE score (n = 65)^b^	46.3(16.4)
ANZROD% (n = 65)^b^	3.4(6.5)
Left ventricular ejection fraction (n = 95) ^b^	55.1(13.4)
CPBT in minutes (n = 95)b	105.8(39.5)
Aortic cross clamp time in minutes (n = 95)^b^	80.7(28.7)
ICU length of stay (days)^b^	3(2)
Total hospital length of stay (days)^b^	11(5)
Discharge destination^a^	
Home/Local accommodation/Rehab	90/6/5
Chest pain at rest (n = 97)^a^	
NRS 0-3/4-6/7-10	78/17/2
Chest pain during coughing (n = 97)^a^	
NRS 0-3/4-6/7-10	38/37/22
Comorbidities^a^	
Hypertension/ Dyslipidaemia /T2DM /Obesity/GORD	71/61/45/43/19
IHD/OSA/Gout/COPD/CKD	19/11/11/7/6

CABG–Coronary artery by-pass graft; AVR–Aortic valve replacement; MVR–Mitral valve replacement; NYHA–New York heart association; APACHE III—Acute physiology and chronic health evaluation III; ANZROD–Australian and New Zealand risk of death; CPBT–Cardiopulmonary by-pass time; ICU–Intensive care unit; NRS–Numerical rating scale; T2DM–Type 2 diabetes mellitus; GORD–Gastro-oesophageal reflux disease; OSA–Obstructive sleep apnoea; CKD–Chronic kidney disease; IHD–Ischaemic heart disease; COPD–Chronic obstructive pulmonary disease.

^a^ Data presented as frequency (%).

^b^ Data presented as mean (standard deviation).

### Changes in lung function, handgrip strength and health-related quality of life

Prior to surgery, patients exhibited 81–98% of their predicted mean FEV_1_, FVC and FEV_1_/FVC and normal lung function patterns. At the time of physiotherapy discharge, most patients developed respiratory profiles that were indicative of restrictive patterns (Table 2). At the time of physiotherapy discharge, FEV_1_, FVC, PEFR and HGS values were significantly reduced by 41%, 37%, 39% and 15% respectively, compared to the pre-operative values (Table 2). For HRQoL, PCS was significantly reduced by 23% following surgery while MCS was similar between pre-operative and physiotherapy discharge values (Table 2).

**Table 2:** Pre-operative and physiotherapy discharge values including change, for handgrip strength, lung function and health-related quality of life of participants.

	Pre-operative [Mean (SD)]		Physiotherapy discharge [Mean (SD)]			
	Actual	%Pred	Actual	%Pred	p-value	Change (%)
FEV_1_(L)	2.46(0.65)	81.6(18.2)	1.43(0.46)	47.3(12.8)	<0.001	-41.10(13.55)
FVC(L)	3.24(0.81)	82.6(17.6)	2.01(0.62)	51.4(12.7)	<0.001	-36.67(14.41)
FEV_1_/FVC	0.76(0.08)	98.6(10.7)	0.71(0.08)	91.7(9.9)	<0.001	
PEFR(L/sec)	6.99(1.95)		4.15(1.53)		<0.001	-38.95(19.19)
HGS(kg)	40.1(10.4)		33.9(10.0)		<0.001	-15.20(13.59)
HRQoL-PCS	41.0(11.1)		31.7(9.4)		<0.001	-17.77(30.50)
HRQoL-MCS	51.0(10.0)		49.7(10.8)		0.22	-0.36(24.33)

FEV_1_ –Forced expiratory volume in one second; FVC–Forced vital capacity; PEFR–Peak expiratory flow rate; HGS–Handgrip strength; HRQoL–Health-related quality of life; PCS–Physical component score; MCS–Mental component score; Change–pre-operative to physiotherapy discharge change in HGS, lung function and HRQoL; %Pred–Percentage of predicted value; p-value–significance level.

### Relationships between handgrip strength, lung function and health-related quality of life

Pre-operatively, significant moderate correlations were identified between HGS and FEV_1_, FVC and PEFR, while moderate correlations were identified between pre-operative HGS and FEV_1_, FVC and PEFR assessed at physiotherapy discharge (Table 3). Significant moderate correlations were identified between HGS, and FEV_1_, and FVC and PEFR at the time of physiotherapy discharge (Table 3). However, no significant relationships were identified between HGS and HRQoL components, pre-operatively and at physiotherapy discharge (Table 3).

**Table 3:** Relationships between handgrip strength, and lung function and health-related quality of life.

	All (n = 101)	
Variables	r-value	p-value
**Pre vs. Pre**		
HGS–FEV_1_	0.52	<0.001
HGS–FVC	0.52	<0.001
HGS–PEFR	0.52	<0.001
HGS–HRQoL-PCS	0.04	0.68
HGS–HRQoL-MCS	0.11	0.29
**Pre vs. Discharge**		
HGS–FEV_1_	0.38	<0.001
HGS–FVC	0.34	<0.001
HGS–PEFR	0.36	<0.001
HGS–HRQoL-PCS	0.02	0.88
HGS–HRQoL-MCS	0.12	0.25
**Discharge vs. Discharge**		
HGS–FEV_1_	0.50	<0.001
HGS–FVC	0.47	<0.001
HGS–PEFR	0.43	<0.001
HGS–HRQoL-PCS	-0.01	0.92
HGS–HRQoL-MCS	0.11	0.27

FEV_1_ –Forced expiratory volume in one second; FVC–Forced vital capacity; PEFR–Peak expiratory flow rate; HGS–Handgrip strength; HRQoL–Health-related quality of life; PCS–Physical component score; MCS–Mental component score; Pre–Pre-operative; Discharge–Physiotherapy Discharge; r-value–Pearson correlation coefficient; p-value–significance level.

### Prediction of lung function and health-related quality of life

Pre-operatively, height and HGS were the most significant predictors of lung function with height contributing 28–35% and HGS contributing 26–38% of the variance in FEV_1_, FVC and PEFR (Table 4). Other variables such as sex and comorbidities contributed significantly to the prediction of pre-operative lung function with these explaining an additional 2–17% of the variance (Table 4). Conversely, pre-operative HGS was not a significant predictor of pre-operative HRQoL (Table 4). At the time of physiotherapy discharge, lung function and HRQoL assessments were predicted by their respective pre-operative values and/or various peri-operative variables including pain during coughing (Table 4). Significant predictors of pre-operative to physiotherapy discharge changes in lung function and HRQoL variables included the respective pre-operative lung function and HRQoL variables, as well as chest pain during coughing, left ventricular ejection fraction, age, presence of chronic obstructive pulmonary disease and aortic cross clamp time (Table 4).

**Table 4:** Regression variables for lung function and health-related quality of life using handgrip strength and other co-variates.

Pre-operative variables	Predictors	B	SE_B_	Adj R^2^	p-value
FEV_1_	Pre-op HGS	0.021	0.006	0.264	<0.001
	Height	2.904	0.680	0.345	<0.001
	Comorbidity- Hypertension	0.255	0.113	0.363	0.026
	Comorbidity-Chronic Kidney Disease	0.434	0.218	0.387	0.049
FVC	Height	3.970	0.868	0.282	<0.001
	Pre-op HGS	0.027	0.007	0.375	<0.001
	Comorbidity- Hypertension	0.299	0.145	0.395	0.043
PEFR	Pre-op HGS	0.077	0.016	0.264	<0.001
	Comorbidity-IHD	1.652	0.378	0.351	<0.001
	Sex	-1.605	0.440	0.411	<0.001
	Comorbidity-Obesity	0.675	0.298	0.435	0.026
HRQoL-PCS	Body Mass Index	-0.556	0.165	0.089	0.001
	Comorbidity- Gout	-7.780	3.458	0.120	0.027
	Comorbidity- Dyslipidaemia	4.467	2.152	0.150	0.041
**Physiotherapy discharge variables**					
FEV1	Pre-op FEV_1_	0.451	0.053	0.417	<0.001
	PainCough	-0.34	0.11	0.472	0.004
	Comorbidity-Dyslipidaemia	-0.161	0.070	0.496	0.024
FVC	Pre-op FVC	0.473	0.055	0.425	<0.001
	PainCough	-0.043	0.16	0.470	0.008
	Comorbidity-Dyslipidaemia	-0.218	0.095	0.495	0.024
PEFR	Pre-op PEFR	0.434	0.065	0.264	<0.001
	PainCough	-0.135	0.042	0.314	0.002
	Employment status	0.483	0.136	0.360	<0.001
	Comorbidity-COPD	1.420	0.472	0.400	0.003
	Body Mass Index	0.041	0.020	0.423	0.040
HRQoL-PCS	Ethnicity	-6.506	1.834	0.112	<0.001
	PainCough	-0.909	0.302	0.186	0.003
HRQoL-MCS	Pre-op MCS	0.474	0.096	0.228	<0.001
	PainCough	-1.097	0.326	0.308	0.001
**Pre-operative to physiotherapy discharge changes**					
FEV_1_	Pre-op FEV_1_	-6.714	2.081	0.095	0.002
	LVEF	-0.267	0.100	0.167	0.009
	PainCough	-1.143	0.460	0.216	0.015
FVC	Pre-op FVC	-6.656	1.663	0.145	<0.001
	LVEF	-0.296	0.104	0.219	0.006
	PainCough	-0.986	0.480	0.249	0.043
PEFR	Pre-op PEFR	-2.178	0.968	0.063	0.027
	Age	-0.453	0.155	0.113	0.005
	Comorbidity-COPD	-13.527	6.619	0.146	0.044
	PainCough	-1.243	0.618	177	0.048
HRQoL-PCS	Pre-op PCS	-1.894	0.222	0.383	<0.001
	Age	-0.668	0.206	0.431	0.002
	PainCough	-2.642	0.840	0.486	0.002
HRQoL-MCS	Pre-op MCS	-1.227	0.223	0.204	<0.001
	PainCough	-1.910	0.776	0.255	0.016
	ACXT	-0.190	0.081	0.294	0.020

FEV_1_ –Forced expiratory volume in one second; FVC–Forced vital capacity; PEFR–Peak expiratory flow rate; HGS–Handgrip strength; HRQoL–Health-related quality of life; PCS–Physical component score; MCS–Mental component score; IHD–Ischaemic heart disease; COPD–Chronic obstructive pulmonary disease; CPBT–Cardiopulmonary by-pass time; PainCough–Chest pain during coughing, ACXT–Aortic cross-clamp time; LVEF—Left ventricular ejection fraction. B–Unstandardized coefficient; SB_E_−Standard error of the coefficient.

## Discussion

This study demonstrated that cardiac surgery resulted in significant reductions in lung function, HGS and the physical component of HRQoL at the time of physiotherapy discharge. Significant but moderate correlations were identified between HGS and lung function variables prior to and following cardiac surgery with HGS a significant predictor of pre-operative lung function. Despite this association, HGS was not a predictor of lung function and HRQoL at physiotherapy discharge with other variables contributing significantly to pre-operative to physiotherapy discharge changes as well as physiotherapy discharge assessments. Collectively, these results indicated that cardiac surgery significantly impaired patient’s lung function, PCS and HGS with HGS being of limited use in predicting lung function and HRQoL at the time of physiotherapy discharge.

The observed, reduced HGS at physiotherapy discharge, which corroborates the study of da Silva et al. [6], could be explained by the effect of inflammation-induced, muscle proteolysis described in post-cardiac surgical patients [8, 27]. However, our findings conflicted with the study by Fu et al. [12], which may be linked to their inclusion of a healthier population with lesser proportions of pre-existing comorbidities (hypertension, diabetes mellitus and dyslipidaemia) among their participants compared to the current study. Further research is needed to confirm our finding given the previously reported association between poor HGS recovery and greater risk of complications within 30 days post-cardiac surgery [12]. The identified reduction in lung function at physiotherapy discharge is similar to previous reports [5, 13]. This finding may be due to previously reported catabolic effects of inflammatory markers on respiratory muscles and surgical-related factors such as general anaesthesia and altered chestwall configurations on lung expansion [7, 28, 29].

Assessment of HRQoL at physiotherapy discharge indicated a reduction in PCS but not MCS. This is not surprising given that the combined reductions in HGS and lung function as well as lower pre-operative PCS reported by these patients (less than population mean score of 50) [22], likely contributed to their reduced post-operative PCS [30]. Non-significant change in MCS observed between pre-operative and physiotherapy discharge assessments could be as a result of the higher pre-operative mean MCS values (greater than population mean score of 50) of the study participants [22]. Further, it is possible that there was an absence of delirium and cognitive decline in this patient group as compared to previously reported studies involving post-cardiac surgical patients [30, 31].

A key finding of the current study was the significant but moderate correlations between HGS and lung function at pre-operation and physiotherapy discharge. To the best of our knowledge, this is first study to investigate these relationships amongst patients undergoing elective cardiac surgery, with previous studies examining these associations primarily in patients with COPD and diabetes [15]. The current finding aligns with the previously identified link between peripheral (assessed by HGS) and respiratory muscle strength in healthy [32] and non-cardiac populations [33], which is a partial determinant of lung function [34]. However, this link between HGS and lung function may have been weakened by post-operative mechanical factors, such as decreased chest expansion and uncoordinated thoracic wall movements impacting on lung function [35].

Whilst the current study identified significant, pre-operative ability of HGS on lung function in cardiac surgical patients, it is currently not recommended that clinicians use HGS as a prognostic tool for lung function and HRQoL. Despite previous studies which reported HGS as a predictor for lung function and HRQoL in healthy [36, 37] and unhealthy populations [38], it is possible that during this acute recovery, there are other stronger factors influencing lung function and HRQoL compared to HGS. For example, chest pain during coughing, as shown in this study, could impact lung function and HRQoL by leading to avoidance of deep breathing exercises, poor lung expansion, immobilisation, further deconditioning and social isolation [13, 39]. In addition, the inability of HGS to predict HRQoL pre-operatively and/or physiotherapy discharge could be explained by its isolated nature, with HRQoL assessment being more holistic as it considers the impact of cardiac disease on patients’ overall well-being during their daily activities [22]. The inconsistent, or lack of, predictive ability of HGS for lung function and HRQoL in cardiac surgical patients highlights the limited potential of HGS as an indirect marker for use by clinicians during post-cardiac surgical in-hospital recovery.

Overall, the current study has identified HGS as an irrelevant tool for predicting lung function and HRQoL at an acute stage of post-cardiac recovery, where inflammatory biomarkers and surgical-related factors may have stronger influence on the activities of the body systems [7, 8, 13]. Further studies may investigate the ability of HGS in predicting lung function and HRQoL during long-term recovery given that the effects of inflammation and other operative factors such as sternotomy-related pain may be persistent after cardiac surgery [27].

### Clinical implications

Although HGS was significantly correlated with lung function prior to and at physiotherapy discharge after cardiac surgery, HGS may be of limited use by clinicians to monitor lung function in acute cardiac surgical patients, especially in busy clinical settings. Continuous assessment of HGS, lung function and HRQoL could be beneficial in monitoring patient recovery post-cardiac discharge.

### Limitations

While others have reported changes in HGS, lung function and HRQoL at various times following cardiac surgery [4, 6, 13], this study was the first to examine the changes in cardiac surgical patients and the ability of HGS to predict lung function and HRQoL in patients undergoing elective cardiac surgery. The current study involved a large sample size with pre-operative and physiotherapy discharge assessments conducted using standardised protocols, a known weakness of prior work [15]. Despite these strengths, there were some limitations that need to be considered for future studies. Patients in this study were predominantly Caucasians who were recruited from one regional area. Therefore, the generalisability of results to other populations worldwide remains to be confirmed. Further, patients were healthier than those in prior studies [3, 4, 6] and were serviced predominantly by a single surgical team that may have impacted on their acute recovery. Future studies across multiple centres with variable patient health status and surgical team configurations are needed to elaborate these findings.

## Conclusion

Cardiac surgery significantly reduced HGS, lung function and the physical component of HRQoL in adults with cardiac disease during the acute recovery period. Despite HGS being positively associated with lung function pre-operatively and at physiotherapy discharge, HGS may be a poor indicator of acute operative changes in lung function and HRQoL in cardiac surgical patients. Future research may determine the suitability of HGS to assess lung function and HRQoL during chronic stages of recovery following cardiac surgery.
